# Extrapolation Yields Painting, Probability, and Predictions

**DOI:** 10.3201/eid2301.AC2301

**Published:** 2017-01

**Authors:** Byron Breedlove, Martin I. Meltzer

**Affiliations:** Author affiliation: Centers for Disease Control and Prevention, Atlanta, Georgia, USA

**Keywords:** art science connection, emerging infectious diseases, art and medicine, about the cover, infectious diseases, extrapolation yields painting, probability, and predictions, Daniel Bernoulli, Robert Ross, Crockett Johnson, Mystic Hexagon, Blaise Pascal, modeling, probability theory, public health

**Figure:**
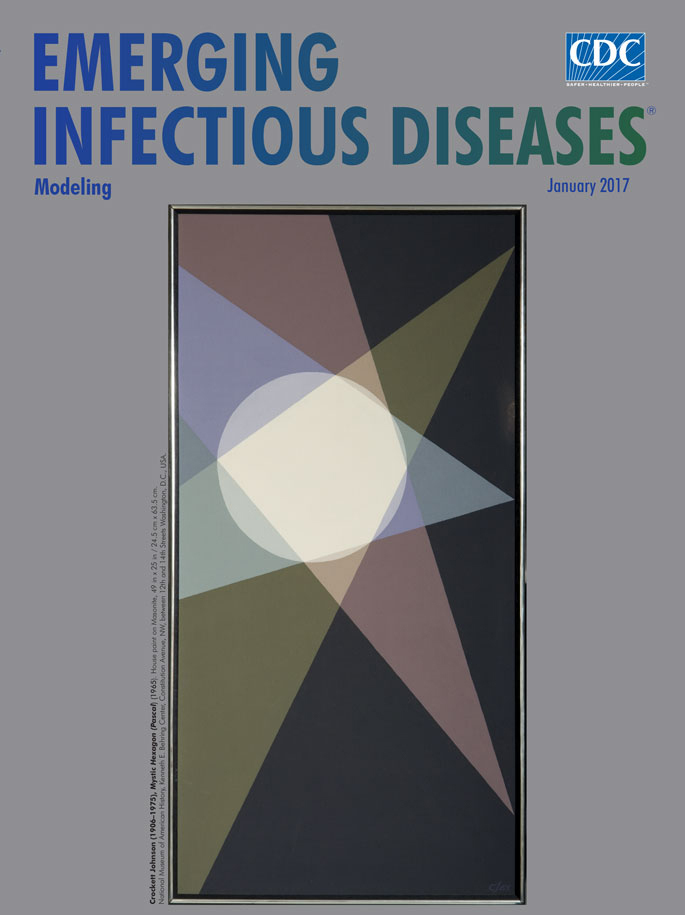
**Crockett Johnson (1906–1975), Mystic Hexagon (Pascal) (1965). House paint on Masonite, 49 in × 25 in/24.5 cm × 63.5 cm.** National Museum of American History, Kenneth E. Behring Center, Constitution Avenue, NW, between 12th and 14th Streets Washington, DC, USA.

“. . . in a matter which so closely concerns the wellbeing of the human race, no decision shall be made without all the knowledge which a little analysis and calculation can provide.”

—Daniel Bernoulli, 1760

According to the National Museum of American History, “Inspired by the allure of the space age, many Americans of the 1960s took great interest in mathematics and science.” Included among these was Crockett Johnson, a well-known cartoonist, book illustrator, and children’s author best remembered for his *Harold and the Purple Crayon* series.

From 1965 until his death in 1975, Johnson painted what he described as “a series of romantic tributes to the great geometric mathematicians from Pythagoras on up.” Initially, Johnson drew inspiration from figures he found in James R. Newman’s book *The World of Mathematics* (1956) and other mathematics books but later began to develop his own geometric constructions. He completed more than 100 of these distinctive paintings of layered, precise geometrical shapes during the last decade of his life.

Critics and art historians have noted that Johnson showed little interest in the technical details of painting. Eschewing convention, Johnson instead preferred to use house paints from a local hardware store and to paint on the rough side of small pieces of Masonite instead of canvas—though he did on occasion both use the smoother side and complete some larger works. Although other contemporary painters such as such as Piet Mondrian, Josef Albers, Alexander Calder, Richard Anuszkiewicz, and Ad Reinhardt (who was a close friend) also used mathematical ideas and geometric shapes, Johnson differed from them in that he linked his geometric paintings with specific mathematicians and he delved into researching and understanding the mathematical ideas that he found inspiring.

Among the earliest of these paintings is this month’s cover art, *Mystic Hexagon* (*Pascal*), which Johnson based on a theorem devised by 16-year-old Blaise Pascal in 1640. In essence, Pascal had postulated that if the opposite sides of an irregular hexagon inscribed in a circle are extended, they meet in 3 points that lie on a straight line. In his depiction of Pascal’s work, Johnson positioned the circle and cream-colored hexagon near the center of the painting. Overlapping wedges of green, blue, and gray form the different pairs of lines. He did not paint the line that would serve to join the 3 intersections (now dubbed the Pascal Line), but the right edge of the painting fulfills that function.

Pascal, like Johnson, was intrigued by numbers, and he made notable contributions to mathematics and science. He is credited with laying the foundation for probability theory through a series of letters he exchanged with Pierre de Fermat. The pair pondered a problem related to expected outcomes in a dice game that vexed an acquaintance who gambled professionally. That correspondence is credited with developing a fundamental theory of probability—the branch of mathematics concerned with analyzing random, or seemingly random, phenomena—with its roots in Pascal’s “Treatise on the Arithmetical Triangle.”

Similar to Pascal’s geometrical extrapolations as depicted in Johnson’s painting, mathematical extrapolations of data have long provided essential information to aid public health officials with decision making. An early example is that of Daniel Bernoulli, who in 1766 used the then relatively new method of calculus to estimate that smallpox elimination via routine vaccination would reduce the risk of death by age 25 years from ≈57% to 50%. Ronald Ross’s model on malaria transmission, first introduced in 2 reports published in 1908 and 1911, is a particularly important example of such modeling for public health decision making. Versions of that model are still used today to inform critical public health decision making regarding malaria control.

Today, mathematical models have become essential tools for public health officials, providing estimates of disease burden, potential impact of interventions, and duration of disease outbreaks. They are particularly useful in situations for which little or no data exist, such as estimates of number of cases of disease in the future, or potential impact (benefit) of a yet-to-be-licensed vaccine. In such situations, mathematical modelers typically use data from different sources, along with assumptions about the underlying transmission, to build (or extrapolate) models to provide estimates for the current problem. Such mathematical models have, with the advent of more powerful and cheap computing capabilities, become ever more diverse in methods and degrees of complexity. Mathematical models of infectious disease can now range from the simple, such as the two-dimensional representation found in Johnson’s painting, to large multidimensional models that simulate the daily contacts between individuals within a community and the resultant risk for onward transmission of infectious disease.
